# Assessment of clinical continuity strategies offered by dual-degree training programs in the USA

**DOI:** 10.1017/cts.2022.454

**Published:** 2022-08-26

**Authors:** Samantha E. Spellicy, Elinor C. Mannon, Audra N. Iness, Hanna L. Erickson, Mariam B. Camacho, Abhik Banerjee, Jillian Liu, Alex Adami, Neal L. Weintraub

**Affiliations:** 1 Medical College of Georgia, University System of Georgia MD-PhD Program, Augusta, GA, USA; 2 American Physician Scientists Association, Westford, MA, USA; 3 Department of Pediatrics, Baylor College of Medicine, Pediatrician Scientist Training and Development Program, Houston, TX, USA; 4 University of Illinois College of Medicine at Urbana, MD-PhD Program, Urbana, IL, USA; 5 University of Southern California and California Institute of Technology, MD-PhD Program, Los Angeles, CA, USA; 6 The Ohio State University Medical Scientist Training Program, Columbus, OH, USA; 7 Department of Medicine, University of Connecticut, Farmington, CT, USA; 8 Medical College of Georgia, Vascular Biology Center, Augusta, GA, USA

**Keywords:** Physician-Scientist, clinical continuity, clinical training, MD-PhD, DO-PhD, medical education

## Abstract

**Background:**

Integration of clinical skills during graduate training in dual-degree programs remains a challenge. The present study investigated the availability and self-perceived efficacy of clinical continuity strategies for dual-degree trainees preparing for clinical training.

**Methods:**

Survey participants were MD/DO-PhD students enrolled in dual-degree-granting institutions in the USA. The response rate was 95% of 73 unique institutions surveyed, representing 56% of the 124 MD-PhD and 7 DO-PhD recognized training programs. Respondents were asked to indicate the availability and self-perceived efficacy of each strategy.

**Results:**

Reported available clinical continuity strategies included clinical volunteering (95.6%), medical grand rounds (86.9%), mentored clinical experiences (84.2%), standardized patients/ practice Objective Structured Clinical Examinations (OSCEs) (70.3%), clinical case reviews (45.9%), clinical journal clubs (38.3%), and preclinical courses/review sessions (37.2%). Trainees rated standardized patients (*µ* = 6.98 ± 0.356), mentored clinical experiences (*µ* = 6.94 ± 0.301), clinical skills review sessions (*µ* = 6.89 ± 0.384), preclinical courses/review sessions (µ = 6.74 ± 0.482), and clinical volunteering (*µ* = 6.60 ± 0.369), significantly (*p* < 0.050) higher than clinical case review (*µ* = 5.34 ± 0.412), clinical journal club (*µ* = 4.75 ± 0.498), and medicine grand rounds (*µ* = 4.45 ± 0.377). Further, 84.4% of respondents stated they would be willing to devote at least 0.5–1 hour per week to clinical continuity opportunities during graduate training.

**Conclusion:**

Less than half of the institutions surveyed offered strategies perceived as the most efficacious in preparing trainees for clinical reentry, such as clinical skills review sessions. Broader implementation of these strategies could help better prepare dual-degree students for their return to clinical training.

## Introduction

Physician-scientists, as defined by the National Institutes of Health (NIH) Physician-Scientists Workforce Working Group (PSW-WG), are scientists with professional degrees who have training in clinical care and are engaged in independent biomedical research. This workforce, which currently totals approximately 9,000 individuals (2008–2012), is uniquely positioned to conduct innovative basic, clinical, and population-based research [1]. These individuals have been crucial in catalyzing scientific and clinical advancements which have shaped the practice of medicine and healthcare in our country. Physician-scientists and their teams were essential in developing the Pfizer, Moderna, and AstraZeneca COVID-19 vaccines, respectively [2,3], and more than half of Nobel Prizes in Physiology or Medicine from 1997 to 2013 were awarded to physician-scientists or teams with at least one MD-PhD [4].

Physician-scientists can pursue several different training pathways, the most traditional of which is a combined dual-degree program in which students receive both clinical and graduate research training. One historical challenge of these dual-degree training programs is termed the “major chasm,” which refers to the lack of integration of clinical skills during the graduate training phases [5]. While dual-degree trainees generally begin the preclinical curriculum at the same time as their MD or DO colleagues, dual-degree trainees typically take a 3–5 year leave of absence from medical school to complete the formal requirements of their PhD program [6]. Reports on the effects of this leave of absence on dual-degree student clinical performance have varied. Some studies have shown that this gap can lead to a lack of confidence in clinical skills compared to their colleagues, especially regarding doctor-patient communication [7]. Additionally, a quantitative comparison at one institution found that MD-PhD students had significantly lower overall Objective Structured Clinical Examination (OSCE) scores than their MD-only colleagues and, in particular, lower scores on cardiovascular and pulmonary OSCE stations [8]. While the question of quantifiable clinical performance differences between dual-degree students and MD/DO-only students remains unanswered, other non-quantifiable elements related to lack of preparedness for clinical reentry should also be considered. For example, isolation from respective cohorts and lack of mentorship during these graduate and clinical transition phases has been cited as a reoccurring theme in MD-PhD trainee attrition [9]. Directed strategies should be implemented by dual-degree training programs to achieve a more seamless transition between the graduate and clinical training phases. These strategies would serve to reinforce physician-scientist identity and clinical preparedness, resulting in a more integrated physician-scientist training pathway as opposed to discrete silos.

While students often pursue their own strategies to help facilitate the transition from research to clinical training [10], there is a need for dual-degree programs to identify the best strategies to support maintenance of clinical skills during PhD training. To address this “major chasm,” many institutions have implemented curricula to provide clinical continuity to dual-degree students during their PhD training. Reported outcomes of these clinical continuity programs typically reveal no difference in performance between dual-degree trainees and their MD or DO counterparts, suggesting that such programs can successfully prepare dual-degree trainees for returning to medical school clerkships [11]. For example, one study reported no difference in National Board of Medical Examiners (NBME) Subject Exam performance, clerkship scores, history-taking skills, or communication skills of returning dual-degree students compared to MD-only students following a refresher course [12]. In addition to refresher courses, other common components of these curricula include physical exam skills review sessions [5,13], practice OSCEs [5,13], history-taking in clinical or hospital settings [5,13], clinical case discussions [11,13], student-run medical clinics [5], and introductory courses on clinical workflow including electronic medical records [11,13]. These activities can occur in the weeks prior to starting clerkships [5,13,14], or throughout PhD training [11]. Surveying the websites of United States MD-PhD programs revealed that all programs have either a formal or recommended clinical reentry curriculum for students [11]. However, the structure of these curricula is largely unstudied and unstandardized.

The specific aims of this study are to 1) ascertain the prevalence of specific interventions that provide clinical continuity during PhD training for dual-degree trainees; 2) identify which of these interventions trainees feel best prepares them for their transition back into clinical training, and 3) investigate any significant differences in perceived efficacy or availability of clinical continuity strategies across demographic groups. We hypothesized that among the variety of clinical continuity strategies utilized at dual-degree training programs across the United States, a subset of these clinical continuity strategies would be viewed by trainees as more useful for their transition back into clinical training. Lastly, this study also aimed to ascertain dual-degree students’ perceptions of the necessity and utility of these clinical continuity strategies.

## Materials and Methods

### Study Design

This study was reviewed and deemed exempt by the IRB at the Medical College of Georgia at Augusta University (IRBnet ID 1351799-3). Data collection, analysis, and reporting were conducted according to the IRB-reviewed protocol with an opt-in consent construct. Target survey participants were current MD/DO-PhD trainees or recent graduates (within one year of completion) from dual-degree-granting institutions in the USA. The Association of American Medical Colleges (AAMC) and the American Association of Colleges of Osteopathic Medicine (AACOM) list 124 MD-PhD granting institutions and 7 DO-PhD granting institutions, respectively, at the time of the survey [15]. Study participants were recruited through the American Physician Scientists Association’s (APSA) 116 institutional representatives (IRs) representing 73 unique institutions (Supplemental Table 1). IRs were emailed anonymous survey links and were asked to forward the email to dual-degree trainees at their institutions (Supplemental Materials 1). The survey was open from October 2019 through December 2019, and one reminder email was sent to IRs each month that the survey was available. Opt-In participant consent was required on the first page of the survey to proceed and complete the survey (Supplemental Materials 2). The survey consisted of 15 questions from 4 different domains, including demographic information (gender identity, ethnicity), training program information (name of institution, year of training), clinical continuity strategies available to the respondent at their institution (when available and if required), and clinical continuity strategy expectations (perceived efficacy and time willing to dedicate per week to continuity strategies). A list of survey questions can be found in the supplemental information (Supplemental Materials 3).

### Data Collection

Results of the study were collected via an online survey and did not contain any identifying features. Responses were stored in a protected location for analysis.

### Statistical Analysis

Responses were evaluated by predefined exclusion criteria. Specifically, all survey responses from individuals who are not current students or graduates of dual-degree (MD/DO-PhD) programs in the USA were excluded. Data were analyzed using JMP® Version 14 (SAS Institute Inc., Cary, NC, 1989–2019). The perceived efficacy of clinical continuity strategies was compared utilizing one-way ANOVA with Tukey’s multiple comparisons post hoc test. The perceived efficacy of clinical continuity strategies by sex and MSTP status was evaluated using the unpaired, nonparametric Mann-Whitney test. *P < 0.05, **P < 0.001.

## Results

### Responses and Demographics

**Fig. 1.**
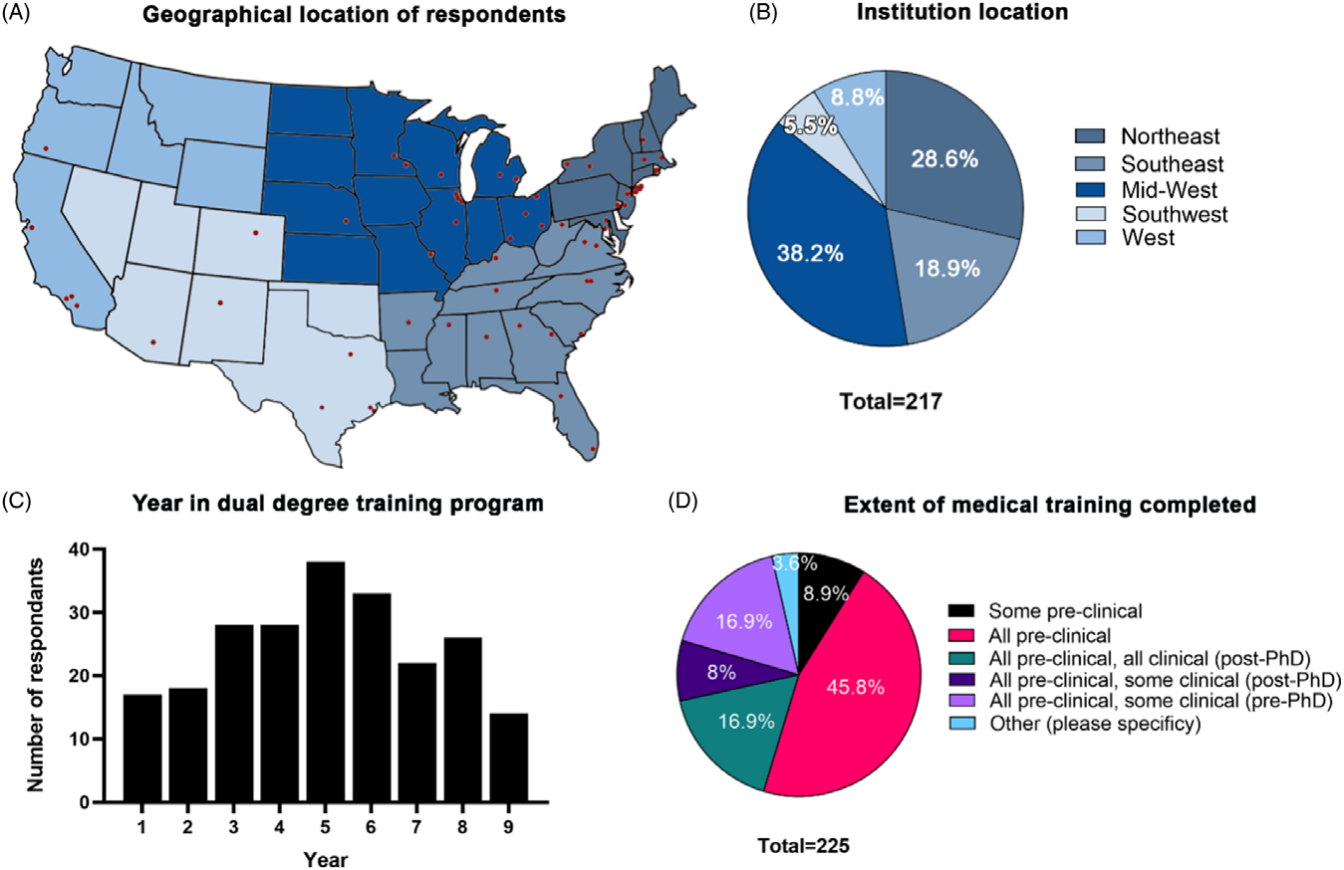
Geographic location and training stage of respondents. **(A)** Map depicting the geographical location of institutions represented in the data set (red dota), as well as regional segmentation (blue gradations). **(B)** Proportion of responses from each of the five designated geographic regions in the United States, Northeast, Southeast, Midwest, Southwest, and West. **(C)** Histogram depicting the distribution of self-reported year in training of dual-degree respondents (*n* = 226). **(D)** Extent of medical training completed at the time of survey.

Out of the students emailed, 226 trainees from 73 unique institutions (87%) responded, representing 56% of the 124 MD-PhD and seven DO-PhD training programs recognized by the AAMC and AACOM (Supplemental Materials Table 1). Respondents were not required to answer all questions to be included in the analysis, which resulted in a variable number of total responses per question. The majority of respondents were located at institutions in the Midwest (38.2%), Northeast (28.6%), and Southeast (18.9%) (Fig. 1A-B). While program structure varied across institutions, 49.3% of respondents were from National Institutes of Health Medical Scientist Training Program (MSTP)-funded MD/PhD programs (Table 1), and responses to the current year of dual-degree training followed a normal distribution (Fig. 1C). Additionally, the majority of respondents (87.6%) had completed all preclinical coursework at the time of the survey, with 16.9% completing all clinical (post-PhD), 8% completing some clinical (post-PhD), and 16.9% completing some clinical (pre-PhD) coursework (Fig. 1D). Of respondents, 120 (53.1%) self-identified as female, 103 (45.5%) self-identified as male, 1 (0.4%) identified as nonbinary, and 2 (0.8%) preferred not to answer. Thirty-six (17.2%) respondents self-reported as an ethnicity that fall under the AAMC’s definition of underrepresented in medicine (UiM) or racial or ethnic groups that are underrepresented in the medical profession relative to their numbers in the general population [16] (Table 1). In accordance with recent MD-PhD sex, race, and ethnicity outcomes studies, respondents were counted as UiM if they indicated one or more of the following groups for their ethnicity: American Indian or Alaska Native, Black or African American, Hispanic or Latina of Spanish origin, and Native Hawaiian or Other Pacific Islander [17]. Lastly, the majority of programs scheduled the graduate phase of training either after completion of the second preclinical (M2) year (78%) or after completion of some clinical (M3) clerkships (12%) (Table 1).

**Table 1.** Demographic data of respondents

Variable	Total
	(*n* = 226)
**Self-Identified Gender**	
*Female*	120 (53.1%)
*Male*	103 (45.5%)
*Non-Binary*	1 (0.4%)
*Prefer not to answer*	2 (0.8%)
**Self-Identified Ethnicity**	
*Ashkenazi Jewish*	1 (0.5%)
*Asian/ Pacific Islander*	43 (19%)
*Black or African American*	14 (6%)
*Caribbean*	1 (0.5%)
*European*	1(0.5%)
*Hispanic or Latinx*	15 (7%)
*Jewish*	1 (0.5%)
*Korean and White*	1 (0.5%)
*Middle Eastern*	1 (0.5%)
*Mixed*	2 (1%)
*North African/ Middle Eastern*	1 (0.5%)
*West Indian*	1 (0.5%)
*White*	132 (59%)
*White and Hispanic or Latinx*	1 (0.5%)
*White and Native American*	1 (0.5%)
*White, Latinx, pacific islander*	1 (0.5%)
*Prefer not to answer*	8 (4%)
**Underrepresented in Medicine (UiM)**	
*Yes*	36 (17%)
*No*	178 (83%)
NIH Funded (Medical Scientist Training Program-MSTP)	
*Yes*	96 (44.04%)
*No*	122 (55.96%)
**Program Structure - Graduate Phase**	
*Before M1*	1 (1%)
*G1 before M1, G3-4 after M2*	2 (2%)
*After completion of M1*	2 (2%)
*After completion of M2*	79 (78%)
*After completion of some M3 clerkships*	12 (12%)
*After completion of all M3 clerkships*	4 (4%)
*Individualized*	1 (1%)

### Offered Clinical Continuity Strategies

**Fig. 2.**
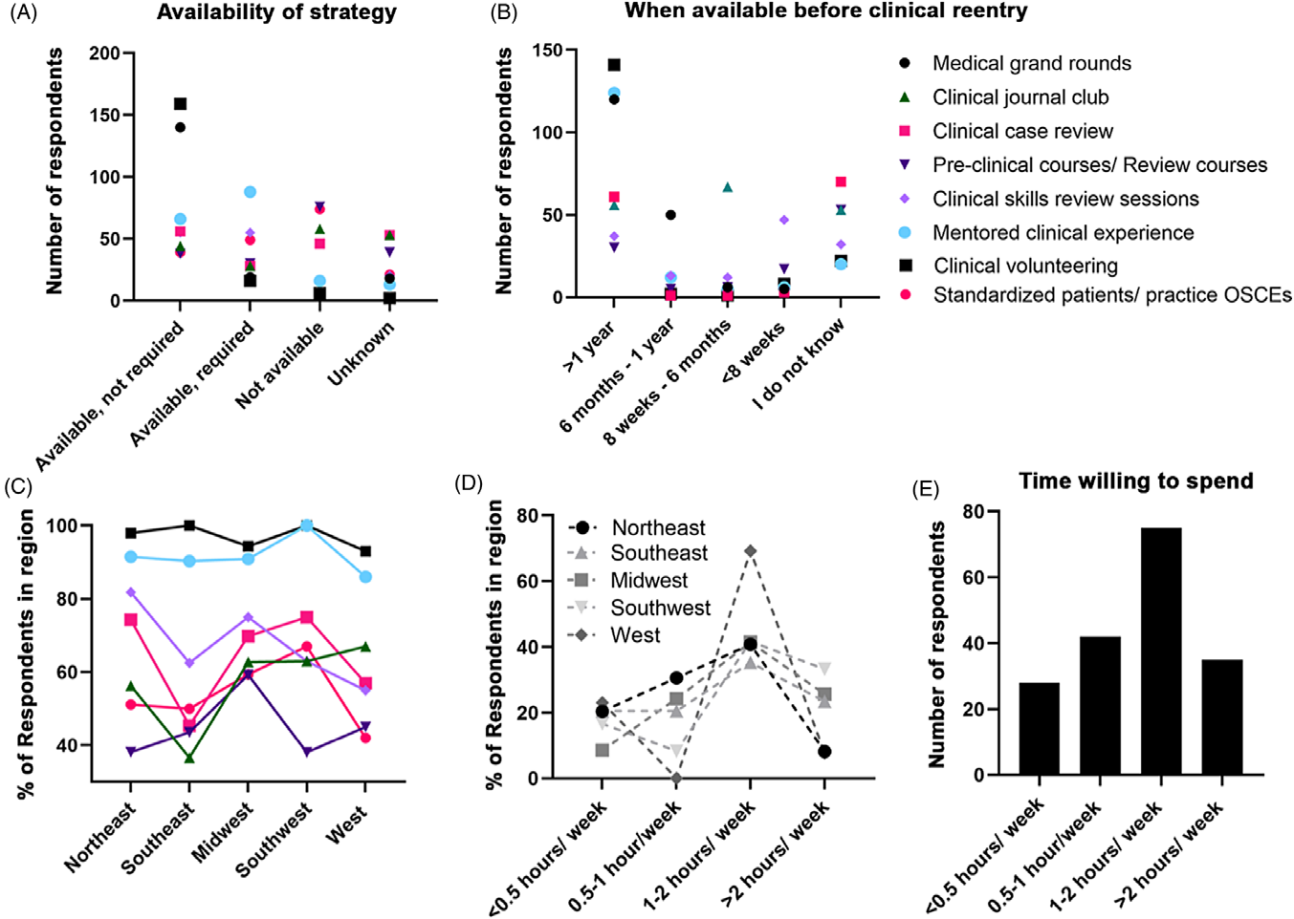
Availability of clinical continuity strategies by dual-degree training program and region. **(A)** The availability and requirement status (available -not required, available- required, not required, or unknown, *x*-axis) of clinical continuity strategies by the number of respondents (*y*-axis). **(B)** Timing of clinical continuity strategies offered by dual-degree training programs (*x*-axis) by number of respondents (*y*-axis)**. (C)** Availability of clinical continuity strategies in each geographic region (*x*-axis) by normalized percentage of responses in each geographical region (*y*-axis). Clinical continuity strategies key applies to **A-C**: medical grand rounds (black circle), clinical journal club (green triangle), clinical case review (pink square), preclinical courses/review courses (dark purple triangle), clinical skills review session (purple diamond), mentored clinical experience (blue circle), clinical volunteering (black square), and standardized patients/practice Objective Structured Clinical Examination (OSCE) (pink circle). **(D)** Amount of time respondents would be willing to dedicate to each clinical continuity strategy per week in each geographical region (*x*-axis) by normalized percentage of respondents in the region (*y*-axis). **(E)** Total amount of time respondents would be willing to dedicate in general to clinical continuity training per week (*x*-axis) by the total number of respondents (*y*-axis).

Respondents were asked to indicate which clinical continuity opportunities were offered by their program. Reported available clinical continuity strategies (either required or not required) included clinical volunteering (95.6%), medical grand rounds (86.9%), mentored clinical experiences (84.2%), standardized patients/practice OSCEs (70.3%), clinical case reviews (45.9%), clinical journal clubs (38.3%), and preclinical courses/review sessions (37.2%). Out of required strategies, mentored clinical experiences were most common (28.1%). Preclinical/review courses and standardized patients were most frequently cited as unavailable at respondents’ institutions (23.2% and 22.6%, respectively). Lastly, 24.2% of trainees reported that they were unaware of the availability of clinical case reviews and clinical journal clubs at their institutions (Fig. 2A).

An important component of strategy availability is the timing of when the strategies are offered with respect to a trainee’s clinical reentry. Strategies most commonly offered remotely to clinical reentry (>1 year) were clinical volunteering (81.0%), mentored clinical experiences (74.3%), and medical grand rounds (66.3%) (Fig. 2B). The majority of trainees indicated they could participate in these strategies to the degree and frequency of their preference throughout their graduate training. Strategies most frequently reported to be only offered within eight weeks of clinical reentry were clinical skills review sessions and preclinical/review courses (Fig. 2B).

The geographical location of institutions may influence the availability of clinical continuity strategies, as certain regions may follow training paradigms traditional to training in the region. Student-orchestrated strategies, such as clinical volunteering and shadowing, were available at the majority of institutions in each region (>90%). Strategies such as clinical case review and clinical journal club, however, while available at the majority of institutions in the Northeast (74.3%, 56.3%), Southwest (75.0%, 63.0%), and Midwest (69.8%, 62.7%), were not as widely available at institutions in the Southeast (45.4%, 36.5%) (Fig. 2C). Respondents in the West were more willing to dedicate 1-2 hours per week to clinical continuity strategies during their research training (69.2%) when compared to respondents in the Northeast (40.8%), Southeast (35.3%), Midwest (41.4%), and Southwest (41.7%) (Fig. 2D). Of interest, 84.4% of respondents, regardless of region, stated that they would be willing to devote at least 0.5–1 hour per week to clinical continuity opportunities during graduate training (Fig. 2E).

### Perceived Efficacy of Offered Clinical Continuity Strategies

**Fig. 3.**
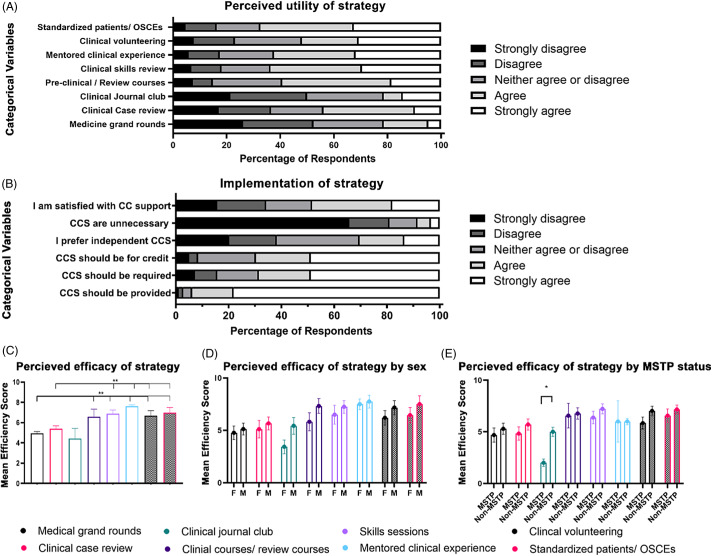
Perceived efficacy of clinical continuity strategies by respondents. All strategies were scored by respondents on a Likert-type scale indicating either strongly disagree (1 and 2), disagree (3 or 4), neither agree nor disagree (5 or 6), agree (7 or 8), and strongly agree (9 or 10). **(A)** The percentage of respondents (*X*-axis) indicating the perceived utility of clinical continuity strategies (CCS) for clinical reentry (*Y*-axis: Categorical variables) offered by dual-degree training programs within each scale designation**. (B)** Rated statements regarding CCS support (*Y*-axis: categorical variables) by the percentage of respondents within each scale designation (*X*-axis). **(C)** Average perceived efficacy of individual CCS (*y*-axis: Mean Efficacy Score), including medical grand rounds (black), clinical journal club (green), clinical case review (pink), preclinical courses/review courses (dark purple), clinical skills review session (light purple), mentored clinical experience (light blue), clinical volunteering (black checkered), and standardized patients/practice OSCE (pink checkered) (*X*-axis, C-E). Data are mean ± standard error. One-way ANOVA with Tukey’s multiple comparisons post hoc was used for statistical analysis. **(D-E)** Average perceived efficacy of CCS (*y*-axis: Mean Efficacy Score) offered by dual-degree training programs, by self-identified gender (**D**, male-M, female-F), and MSTP designation (**E**, MSTP vs. Non-MSTP). Data are mean ± standard error. Unpaired, nonparametric Mann-Whitney test was used for statistical analysis *P < 0.05, **P < 0.001 **C-E.**

To assess the perceived efficacy of the various clinical continuity strategies, respondents were asked to rate the self-perceived efficacy of each strategy on a scale from 1 to 10 (1-completely disagree, 10-completely agree). Ratings were grouped into ranges of strongly disagree (1–2), disagree (3–4), neither agree nor disagree (5–6), agree (7–8), and strongly agree (9–10). Clinical journal clubs, clinical case reviews, and medicine grand rounds had the widest distribution of perceived efficacy responses. In contrast, standardized patients, clinical volunteering, mentored clinical experiences, clinical skills review, and preclinical/ review courses ratings were predominantly toward the top (>6) of the efficacy scale (Fig. 3A). This variability could be due to personal preference or differences in implementation of the strategy at each institution. Analysis revealed trainees rated standardized patients/OSCEs (mean ± SE, *µ* = 6.98 ± 0.356), mentored clinical experiences (*µ* = 6.94 ± 0.301), clinical skills review sessions (*µ* = 6.89 ± 0.384), preclinical courses/review sessions (*µ* = 6.74 ± 0.482), and clinical volunteering (*µ* = 6.60 ± 0.369) significantly higher (*p* < 0.050) than clinical journal club (*µ* = 4.75 ± 0.498), medicine grand rounds (*µ* = 4.45 ± 0.377), and clinical case review (*µ* = 5.34 ± 0.412) (Fig. 3C).

Trainees were also asked to rate their agreement with a series of questions regarding the implementation of clinical continuity strategies. A majority (78.1%) of respondents indicated that they strongly agreed that clinical continuity strategies should be provided by institutions, with 66.9% reporting the highest agreement rating (10). In direct comparison, a majority (65.7%) of respondents strongly disagreed when asked if clinical continuity strategies are unnecessary, with 53.3% assigning it the strongest disagreement rating (1). Of interest, 48.9% of trainees strongly agreed that clinical continuity strategies should be provided for class credit, while 48.8% strongly agreed that they should be required. Strikingly, only 18.0% strongly agreed that they are satisfied with the clinical continuity support at their program, and only 13.5% strongly agreed that they prefer to pursue independent clinical continuity opportunities (Fig. 3B).

Differences in the perceived efficacy of various clinical continuity strategies were also stratified by self-reported sex and NIH-funded status (MSTP vs. Non-MSTP). Responses of “non-binary” and “prefer not to answer” were excluded as the numbers were insufficient within each category to power the analysis. Respondents who self-identified as female reported a trending decrease in perceived efficacy of clinical journal clubs compared to self-reported males (Fig. 3D). Similarly, MSTP students rated clinical journal clubs significantly lower than non-MSTP students (MSTP *µ* = 2.875 ± 0.549 vs. non-MSTP *µ* = 5.500 ± 0.587) (Fig. 3E).

## Discussion

This study assessed the prevalence and variety of clinical continuity strategies provided to dual-degree trainees across the US, as well as the perceived efficacy of these strategies by trainees. It is the first study to our knowledge that assessed regional variability in the utilization of these strategies. The reported most widely available strategies were clinical volunteering experiences and medical grand rounds. These divergent approaches of active patient-centered learning and traditional lecture-style teaching have been widely utilized for years in medical education [18–20]. Previous studies have shown that when lecture formats precede patient interactions, students report better enjoyment, and perform better [21]. Availability of both types of strategies to dual-degree students allows them to choose the preferred format combinations and teaching styles that best accommodates their busy and irregular graduate education schedule. Notably, preclinical or review courses and standardized patients were most frequently cited as unavailable at respondents’ institutions. This was an unexpected finding of the study as review courses are often cited as one of the most efficacious for preparing dual-degree trainees for clinical reentry [12]. These strategies are relatively structured and require more time commitment from trainees, which has implications for their utility closer to clinical reentry. This may explain why these strategies, if offered, were most reported to be available only within eight weeks of clinical reentry.

When considering regional differences in clinical continuity strategies, mentored clinical experiences and clinical shadowing were reported to be available at most institutions regardless of geographical location (Fig. 2C). There were, however, regional differences in the reported availability of other assessed strategies. Namely, while clinical case review was available at the majority of institutions in the Northeast, Midwest, Southwest, and West, it was only reportedly available at a minority of institutions in the Southeast. Moreover, clinical skills review courses and clinical journal clubs were less reportedly employed in the Southeast compared to other regions across the US (Fig. 2C). While organizations such as the AAMC report outcomes data of MD-PhD programs in regard to time to degree, specialty, faculty position, types of post-graduate research, and other metrics stratified by sex, these outcomes are not stratified by region of training [17,22,23]. More detailed regional analysis of trainee outcomes should be included to identify if these differences in availability of clinical continuity strategies and other training paradigms influence the eventual success metric outcomes of MD-PhD or DO-PhD students. This information may also be useful to students applying to dual-degree training programs to help tailor their selection to programs and regions with strategies complementary to their training needs.

Upon assessment of the self-perceived efficacy of clinical continuity strategies in this study, patient-based strategies such as standardized patients, mentored clinical experiences, and clinical volunteering were rated significantly higher than lecture-style strategies such as grand rounds, case reviews, and journal clubs. Of note, strategies that trainees perceived as the most efficacious in preparing trainees for clinical reentry, such as clinical skills review sessions, were reportedly offered at less than half of the institutions surveyed. Respondents also strongly agreed that clinical continuity strategies should be offered at their institutions, and they are currently not satisfied with the available training opportunities. Universal availability of the clinical continuity strategies with the highest efficacy ratings by trainees might help improve the confidence and performance of dual-degree students. Qualitatively evaluating such strategies on performance (i.e., NBME Subject Exam and clerkship grades) and personal satisfaction (i.e., minimizing feelings of remoteness and isolation) would be required to test their efficacy in dual-degree students.

While not assessed in this study, direct mentoring from dual-degree faculty has been reported by students to facilitate reentry into the clinical environment and mitigate feelings of isolation and uncertainty [7]. Lack of mentorship has also been identified as one of the most common self-reported reasons for student attrition from dual-degree programs [9]. These elements relate to an overarching strategy that has been found to be essential to dual-degree student success: “personal development planning.” Through personal development planning, students work with a mentor to create an outline or timeline of which clinical continuity strategies they wish to pursue at what stages and frequency in their graduate training. This allows students to work with programs to implement student-specific adjustments to current strategies [24]. This strategy also allows students to plan for upcoming clinical continuity strategies and seek mentorship in these areas ahead of time. Future studies of dual-degree programs in the USA should include an assessment of formal or informal mentoring opportunities available to trainees and the implementation of formalized personal development planning during their clinical and graduate years.

While previous studies have reported that trainees do not experience differences in transition experiences between graduate and clinical training depending on gender or ethnicity [7], trending differences in perceived efficacy of strategies were noted between students of differing self-reported sex in this study. While not statistically significant, the average perceived efficacy ratings of all strategies were lower in self-identified females than in self-identified males. Future, higher powered studies should be conducted to statistically determine the relevance of this trend. Additionally, MSTP students rated clinical journal clubs as having significantly lower efficacy than non-MSTP students. Understanding this apparent disparity could facilitate strategic implementation to provide equitable clinical continuity regardless of gender or NIH funding status.

Another crucial area of equity investigation is the perceived efficacy of these strategies across populations traditionally underrepresented in medicine (UiM). While this question is of great interest, this study did not have enough statistical power to analyze the differences between UiM and non-UiM respondents. Our inability to garner a significant number of responses from UiM students likely reflects the relatively low number of UiM students currently enrolled in dual-degree programs in the USA. A recent study showed that between 2005 and 2014, less than 10% of MD-PhD graduates were from traditional UiM groups [17]. While the proportion of UiM to non-UiM students has steadily increased over time, from 1.3% in 1975 to 9.8% from 2005 to 2014, it is evident this increase has been insufficient to address the disparities [17].

While this study assessed the availability and perceived efficacy of clinical continuity strategies across the US, there were certain limitations. A perennial challenge for MD/DO-PhD training, and this study concerns the wide variety of sizes represented by the many training programs. Nationally, program matriculating class sizes range from 1–2 to several dozen trainees per year [25]. A program with 100 or more total trainees may find a dedicated course for its trainees to be practical and attainable. At the same time, one with less than 20 may, by necessity, rely on existing resources within a larger school of medicine. The heterogeneity of program structures across the US further makes comparisons challenging. For example, programs in which students complete some or all of their clinical clerkships before their graduate training may find certain strategies like clinical refresher courses or OSCEs unnecessary. As 78% of respondents in this study attended programs with the graduate training scheduled directly following completion of preclinical training (M2), such program heterogeneity is unlikely to have significantly influenced our findings. Still, it may limit generalizability to different program structures. The same is true for comparisons between programs with and without NIH MSTP funding. Factors correlated with MSTP status, such as cohort size and available resources, may impact the perceived efficacy of various strategies.

An additional limitation of the study involves the per-program respondent percentage, as well as the as well as the responding program percentage out of all available programs. Approximately half of the available dual-degree programs were represented by respondents in this study. While this sample provides a preliminary understanding of the clinical continuity strategies offered by dual-degree programs in the USA, larger comprehensive follow-up studies should be performed to obtain a more representative sample of responses. Additionally, within programs, not all current dual-degree trainees responded to the study. This may bias the results toward those who feel more strongly positively or negatively about clinical continuity strategy experiences [26]. Geographic variety is both a strength and a weakness of the present study. We surveyed programs in multiple regions and of multiple sizes, but the conclusions from and comparisons between regions are necessarily limited by sample size (e.g., Southwest and West). However, while individual comparisons by region, size, and environment are challenging, the broad agreement on the necessity of clinical continuity strategies (Fig. 3) demonstrates the need for further study. Another limitation inherent to retrospective survey analysis is the reliance on recall and subjective experiences. Determining whether the variability in responses pertaining to the efficacy of certain strategies was due to intrinsic differences in training preferences versus the actual implementation of strategies at each institution should be ascertained in future studies. There may similarly be bias in terms of who responded to the survey. While broad in geography, trainees from just over half of programs nationally responded, and there may be biases in responding versus nonresponding programs that could limit conclusions. For example, programs with few or no respondents may have trainees more satisfied with their clinical continuity strategies who are consequently less likely to respond. The present work may serve as a pilot study for a more comprehensive assessment, perhaps as a collaboration between APSA and the National Association of MD-PhD Programs. Such a collaboration could encourage participation by both program leadership and trainees themselves.

## Conclusions

Overall, this study provides important insights into trainee perceptions of available clinical continuity strategies. Of note, strategies that were perceived as the most efficacious in preparing trainees for clinical reentry, such as clinical skills review sessions, were offered at less than half of the institutions surveyed. Broader implementation of such strategies may help assuage dual-degree student concerns and improve their preparation for the return to clinical training.

## Supplementary material

For supplementary material accompanying this paper visit https://doi.org/10.1017/cts.2022.454. click here to view supplementary material
