# Supplementary material for: Assessment of clinical continuity strategies offered by dual-degree training programs in the USA
Source: J Clin Transl Sci. 2022 Aug 26;6(1):e116. doi: 10.1017/cts.2022.454 (PMC9549578; doi:10.1017/cts.2022.454)
Supplement: Supplementary file 1 [file S205986612200454Xsup001.docx]

Supplemental Materials

Supplemental Table 1 – Institutions Anonymized

| Institution Anonymized | Region | Raw number | Percentage |  |
| --- | --- | --- | --- | --- |
| A | Midwest | 12 | 5.5% |  |
| B | Northeast | 2 | 0.9% |  |
| C | Northeast | 1 | 0.5% |  |
| D | Northeast | 3 | 1.4% |  |
| E | Northeast | 2 | 0.9% |  |
| F | Northeast | 1 | 0.5% |  |
| G | Southeast | 1 | 0.5% |  |
| H | Southeast | 3 | 1.4% |  |
| I | Southeast | 1 | 0.5% |  |
| J | Northeast | 2 | 0.9% |  |
| K | Northeast | 1 | 0.5% |  |
| L | West | 1 | 0.5% |  |
| M | Midwest | 5 | 2.3% |  |
| N | Southeast | 9 | 4.1% |  |
| O | Southeast | 1 | 0.5% |  |
| P | Midwest | 6 | 2.8% |  |
| Q | Northeast | 1 | 0.5% |  |
| R | Northeast | 9 | 4.1% |  |
| S | Midwest | 5 | 2.3% |  |
| T | Midwest | 5 | 2.3% |  |
| U | Midwest | 2 | 0.9% |  |
| V | West | 3 | 1.4% |  |
| W | Northeast | 1 | 0.5% |  |
| X | Northeast | 1 | 0.5% |  |
| Y | Northeast | 3 | 1.4% |  |
| Z | Midwest | 2 | 0.9% |  |
| AA | Midwest | 1 | 0.5% |  |
| AB | Midwest | 3 | 1.4% |  |
| AC | Northeast | 12 | 5.5% |  |
| AD | West | 1 | 0.5% |  |
| AE | Northeast | 1 | 0.5% |  |
| AF | Northeast | 8 | 3.7% |  |
| AG | Northeast | 1 | 0.5% |  |
| AH | Northeast | 1 | 0.5% |  |
| AJ | Northeast | 1 | 0.5% |  |
| AK | Southeast | 4 | 1.8% |  |
| AL | Southwest | 2 | 0.9% |  |
| AM | Southeast | 4 | 1.8% |  |
| AN | Northeast | 4 | 1.8% |  |
| AO | West | 1 | 0.5% |  |
| AP | Midwest | 1 | 0.5% |  |
| AQ | Midwest | 1 | 0.5% |  |
| AR | West | 2 | 0.9% |  |
| AS | Northeast | 1 | 0.5% |  |
| AT | Southeast | 3 | 1.4% |  |
| AU | Midwest | 2 | 0.9% |  |
| AV | Midwest | 2 | 0.9% |  |
| AW | Southeast | 4 | 1.8% |  |
| AX | Northeast | 2 | 0.9% |  |
| AY | Southeast | 3 | 1.4% |  |
| AZ | Midwest | 1 | 0.5% |  |
| BA | Midwest | 5 | 2.3% |  |
| BB | Southeast | 2 | 0.9% |  |
| BC | Midwest | 3 | 1.4% |  |
| BD | Southwest | 1 | 0.5% |  |
| BE | Southeast | 3 | 1.4% |  |
| BF | West | 2 | 0.9% |  |
| BG | Northeast | 2 | 0.9% |  |
| BH | Northeast | 1 | 0.5% |  |
| BI | West | 10 | 4.6% |  |
| BJ | Southwest | 4 | 1.8% |  |
| BK | Southwest | 2 | 0.9% |  |
| BL | Southwest | 1 | 0.5% |  |
| BM | Southwest | 1 | 0.5% |  |
| BN | Southeast | 2 | 0.9% |  |
| BO | West | 1 | 0.5% |  |
| BP | Midwest | 3 | 1.4% |  |
| BQ | Southeast | 1 | 0.5% |  |
| BR | Midwest | 21 | 9.6% |  |
| BS | Southeast | 1 | 0.5% |  |
| BT | Northeast | 1 | 0.5% |  |
| BU | Northeast | 2 | 0.9% |  |

Supplemental 1- Recruitment Materials

**RECRUITMENT MATERIALS**

For recruitment of survey participants, an email will be sent to elected American Physician Scientist Association (APSA) institutional representatives or trainee members who represent the colleges and universities in which they train.

The recruitment email will read as follows,

Hello,

The American Physician Scientist Association is conducting a research study about clinical continuity strategies employed by dual degree physician-scientist programs across the United States. We are emailing you to ask if you would like to take approximately 7 minutes to complete a survey for this research project. Participation is completely voluntary, and your answers will be anonymous. If you are interested, please click on the link for the survey and additional information.

<https://www.surveymonkey.com/r/APSAclinicalcontinuity>

 If you have any questions, please do not hesitate to contact Samantha Spellicy at

[Samantha.spellicy@physicianscientists.org](mailto:Samantha.spellicy@physicianscientists.org)

Thank you,

American Physician Scientists Association

Supplemental 2- Consent for Participation

Dear American Physician Scientist Association Member,

You are invited to participate in a research project to study clinical continuity strategies of dual degree physician-scientist training programs in the United States because, according to our records, you are currently enrolled in a dual degree program. This research is sponsored by the American Physician Scientist Association (APSA). The survey asks questions about your experience with various optional and mandatory clinical continuity strategies at your institution. It should take you about 7 minutes to complete

Through your participation, we hope to understand the prevalence of specific interventions that provide clinical continuity during the Ph.D. training for dual degree programs. Additionally, we hope that the results of the survey will be useful for identifying which of these interventions trainees feel best prepares them for their transition back into clinical training. We hope to share our results through presentations at meetings, such as APSA’s annual meeting, as well as publish them in a scientific journal.

There are no known risks to you if you decide to participate in this survey. There is no direct benefit to you for participating in this study. The alternative would be not participating in the study. I will not share any information that identifies you with anyone outside my research group, which consists of myself, and various APSA committee members directly involved in the project.

I will do my best to keep your information confidential. All data is stored in a password-protected electronic format.  Email addresses will initially be collected to ensure no duplicates or repeat submissions in the data. To help protect your confidentiality, these email addresses will then be completely dissociated from the accompanying responses, and the data will be aggregated. Additionally, there will be an option to allow APSA to retain your email addresses for possible studies in the future. Allowing retention of your email is completely optional and will have no impact on your survey responses or participation in this current study. The results of this study will be used for scholarly purposes only and may be shared with Augusta University and APSA representatives.

I hope you will take the time to complete this questionnaire; however, if you agree to complete the survey, you are not required to answer all the questions or complete it. Your participation is voluntary, and there is no penalty if you do not participate.  If you have any questions or concerns about completing the questionnaire, about being in this study, or about receiving a summary of my findings, you may contact me by email at [*sspellicy@augusta.edu*](mailto:sspellicy@augusta.edu) or by phone at 301-633-9471.

If you have any questions or concerns about the “rights of research subjects”, you may contact the Augusta University IRB Office at (706) 721-1483.

Sincerely,

Samantha Spellicy

Supplemental 3- Survey Questions

Consent

1. Do you consent to participate in this study? Clicking on the “agree” button below indicates that:

- You have read the above information
- You voluntarily agree to participate
- You are at least 18 years of age
  1. Agree
  2. Disagree

Demographics

1. To which gender do you most identify? (Select one)
   1. Female
   2. Male
   3. Transgender female
   4. Transgender male
   5. Gender variant/non-conforming
   6. Prefer not to answer
   7. Other (please specify)
2. What is your ethnicity? (Select all that apply)
   1. White
   2. Hispanic or Latinx
   3. Black or African American
   4. Native American or American Indian
   5. Asian/Pacific Islander
   6. Other (please specify)
3. How many years of dual-degree training will you have completed at the end of the Fall 2019- Spring 2020 school year?
   1. 1
   2. 2
   3. 3
   4. 4
   5. 5
   6. 6
   7. 7
   8. 8
   9. 9+
4. How much medical training will you have completed at the end of the Fall 2019- Spring 2020 school year?
   1. Some pre-clinical coursework
   2. All pre-clinical coursework
   3. All pre-clinical coursework, some clinical clerkships (pre-PhD)
   4. All pre-clinical coursework, some clinical clerkships (post-PhD)
   5. All pre-clinical coursework, all clinical clerkships (post-PhD)
   6. Other (please specify)
5. How much graduate training will you have completed at the end of the Fall 2019- Spring 2020 school year?
   1. No graduate training
   2. Some graduate training
   3. All of graduate training

Training Program Information

1. What is the name of your institution?
   1. [Drop down list]
2. Is your program…
   1. MD-PhD
   2. DO-PhD
3. When does your program schedule the graduate phase (if multiple options are available, please select the time you chose):
   1. After completion of M1
   2. After completion of M
   3. After completion of some M3 clerkships
   4. After completion of all M3 clerkships
   5. Other (please specify)

Clinical Continuity Strategies

1. For each of the clinical continuity strategies below, please indicate whether they are available and required by your program, available and not required by your program, or not available during your graduate phase training.
   1. Medicine grand rounds
   2. Clinical case review
   3. Clinical journal club
   4. Pre-clinical courses/review courses
   5. Clinical skills review sessions
   6. Mentored clinical experience (i.e., shadowing/preceptorship/longitudinal clerkship)
   7. Clinical volunteering (i.e., at a free/community clinic)
   8. Standardized patients/practice OSCEs
   9. Other (please specify)
2. For each of the clinical continuity strategies below, please indicate when these activities are available during your graduate phase training (i.e., <8 weeks, 8 weeks-6 months, 6 months-1 year, or >1 year before returning to medical school full time).
   1. Medicine grand rounds
   2. Clinical case review
   3. Clinical journal club
   4. Pre-clinical courses/review courses
   5. Clinical skills review sessions
   6. Mentored clinical experience (i.e., shadowing/preceptorship/longitudinal clerkship)
   7. Clinical volunteering (i.e., at a free/community clinic)
   8. Standardized patients/practice OSCEs
3. For each of the clinical continuity strategies below, please rate your confidence in the following statement on a scale of 1-10, with 1 being absolutely disagreed and 10 being absolutely agree. “This clinical continuity strategy prepared me adequately for entry or reentry into clinical clerkships.” If you have not entered or re-entered clinical training or did not participate in a particular activity, please respond N/A.
   1. Medicine grand rounds
   2. Clinical case review
   3. Clinical journal club
   4. Pre-clinical courses/review courses
   5. Clinical skills review sessions
   6. Mentored clinical experience (i.e., shadowing/preceptorship/longitudinal clerkship)
   7. Clinical volunteering (i.e., at a free/community clinic)
   8. Standardized patients/practice OSCEs

Clinical Continuity Expectations

1. What are your expectations for clinical skills continuity resources and programs? Select all that apply
   1. Career exploration
   2. Build confidence in patient encounters
   3. Maintain clinical skills
   4. Inform research
   5. Build camaraderie with clinical teams
   6. Other (please specify)
2. How much time would you be willing to devote to clinical continuity activities during graduate training?
   1. <0.5 hours/week (26 hours/year)
   2. 0.5-1 hour/week (26-52 hours/year)
   3. 1-2 hours/week (52-104 hours/year)
   4. >2 hours/week (>104 hours/year)
3. Please rate your confidence in the following statements on a scale of 1-10, with 1 being absolutely disagree and 10 being absolutely agree.
   1. I am satisfied with the amount of support and resources offered by my program for maintaining clinical continuity skills for entry or reentry into clinical clerkships.
   2. Clinical continuity programs should be provided by dual-degree programs.
   3. Clinical continuity programs should be required by dual-degree programs.
   4. Clinical continuity programs should be offered for credit by dual-degree programs.
   5. I prefer the option to independently pursue opportunities to maintain clinical skills. I do not think it is necessary to participate in clinical continuity programs during clinical training.
